# Evidence-informed policy for tackling adverse climate change effects on health: Linking regional and global assessments of science to catalyse action

**DOI:** 10.1371/journal.pmed.1003719

**Published:** 2021-07-20

**Authors:** Robin Fears, Khairul Annuar B. Abdullah, Claudia Canales-Holzeis, Deoraj Caussy, Andy Haines, Sherilee L. Harper, Jeremy N. McNeil, Johanna Mogwitz, Volker ter Meulen

**Affiliations:** 1 InterAcademy Partnership, Trieste, Italy; 2 MAHSA University, Salangor, Malaysia; 3 Integrated Epidemiology Solutions, Ebene Reduit, Mauritius; 4 Centre on Climate Change and Planetary Health, London School of Hygiene and Tropical Medicine, United Kingdom; 5 University of Alberta, Canada; 6 University of Western Ontario, London, Canada

## Abstract

Robin Fears and co-authors discuss evidence-informed regional and global policy responses to health impacts of climate change.

**Summary points**

- Effective policy making depends on synthesising and improving the use of existing robust scientific evidence, tackling misinformation, and identifying knowledge gaps to be filled by new research.
- A global project organised by the InterAcademy Partnership (IAP) is bringing together evidence from Africa, Asia, the Americas, and Europe to evaluate climate change effects on health and to assess policy priorities for adaptation and mitigation solutions. Project design encouraged inclusivity in assessing research from across disciplines and from diverse geographical and socioeconomic contexts encompassing issues for vulnerable groups (including Indigenous Peoples) and integrating outputs at national, regional, and global levels.
- Coordinated policy development approaches across sectors and regions and integration at national–regional–global levels are essential to understand trade-offs, avoid inadvertent consequences, and capitalise on potential synergies for multiple benefits for health, equity, and environment.
- National priorities must include integrating health actions into national climate adaptation plans and Nationally Determined Contributions (NDCs) under the Paris Agreement. Regional policy action is important to address cross-boundary issues and to build critical mass for quantifying and implementing solutions.
- A focus on human health can catalyse the strengthening of international coherence and commitment to tackling shared climate change challenges. Health must be prioritised in current global policy initiatives, including the United Nations Framework Convention on Climate Change (UNFCC) Conference of the Parties 26 (COP26), UN Convention on Biological Diversity (CBD) Conference of the Parties 15 (COP15), and the UN Food Systems Summit. The scientific and health communities have a key role to help lead efforts by engaging at the science–policy interfaces to address barriers to action.

## Introduction

There is increasing evidence of both direct and indirect adverse effects of climate change on human health worldwide, the latter mediated by disruption in ecological and socioeconomic systems [1]. Many of these effects have been known for some time; yet, despite the accumulating evidence, protecting human health has only recently become a major consideration in global policy discussions on climate change [2]. This increased recognition of health issues, brings new demands for evidence from decision makers and requiring robust scientific data, including on attribution, for knowledge synthesis, and its use to inform policy for health and healthcare for both climate change adaptation and mitigation. The scientific and health communities have important roles, including ensuring that robust evidence is generated, sharing lessons of good practice, and building capacity for action worldwide.

Effective policy responses to the multiple effects of climate change on health require integrating diverse mitigation and adaptation measures. Actions to reduce emissions of greenhouse gases (GHGs) can benefit population health locally and in the near term, such as by reducing exposure to co-emitted air pollutants. These are additional to the environmental and global health benefits that will flow from mitigation as a result of reduced climate hazards and could help, by averting costs of ill health (including impacts on labour productivity) and of health services provision, to offset the costs of tackling climate change. Adaptation actions to support individuals, communities, and governments in coping with unavoidable consequences can reduce the effects of climate change on health. Policies designed to mitigate and adapt to climate change should aim to minimise unintended adverse consequences, assess trade-offs [3], and link to health systems guidance [4]. Developing solutions must be based on the synthesis of available research findings from across multiple disciplines and knowledge systems (including from Indigenous Peoples), while recognising the need to fill critical knowledge gaps with new research and tackle misinformation. There is much more to do in all regions. In the European Union, for example, health co-benefits are now explicitly considered when developing climate change mitigation policies, but their influence on final policy outcomes has been limited [5]. Resilient and innovative responses to climate change require a systems-based approach [1,6] involving coherent and coordinated policy development across all sectors and ensuring “health in all policies.”

One of the problems in using scientific evidence has been the hitherto limited input from low- and middle-income countries (LMICs), resulting in lack of inclusivity in designing research and using research outputs for implementing policy and practice. In this paper, we draw on current work of the InterAcademy Partnership (IAP), the global network of more than 140 academies of science, engineering, and medicine, including evidence from diverse geographical and socioeconomic contexts. We explore issues for the science–policy interfaces in climate change and health, making the case for increasing inclusivity and strengthening capacity in marshalling diverse evidence and perspectives to support climate action. The IAP assessment aims to summarise evidence of the effects of climate change on health and potential solutions within and between regions, taking particular account of the issues for vulnerable groups and the need to respect cultural and other diversity. Furthermore, it aims to stimulate integration of policy and practice at local, regional, and global levels, recognising heterogeneity within and between regions. The conclusions from the project are being discussed with the wider scientific and health communities, with policy makers and other stakeholders.

### Collecting and integrating regional evidence

IAP developed an innovative project involving regional working groups, including experts from the social, biological, health, and physical sciences, constituted by academy networks in Africa (Network of African Science Academies, NASAC), Asia-Pacific (Association of Academies and Societies of Sciences in Asia, AASSA), the Americas (InterAmerican Network of Academies of Sciences, IANAS), and Europe (European Academies’ Science Advisory Council, EASAC); see S1 Text. The EASAC report has been published [7]. The project design is distinctive in terms of the commitment to working with academy-nominated scientists worldwide, including those in LMICs, in the collective effort to analyse challenges and opportunities across disciplines and sectors, connecting the scientific evidence to options for policy development, and conforming to shared academy standards of independence, transparency, and excellence. The objective of scientific excellence is accompanied by an objective of capacity building in the newer or smaller academies. The project design objectives differ from but complement, for example, the Lancet Countdown initiative [2], not seeking specifically to monitor over time the effects of climate change on health but rather to engage science academies in summarising evidence of climate–health linkages for their own countries/regions with a focus on solutions and policy opportunities. The attributes of the innovative project design and the research methodologies to facilitate interregional coordination and engagement at the science–policy interface to inform cohesive policy making are discussed in detail elsewhere for a previous project on food and nutrition security and agriculture [8,9]. The added values of this project design, even when similar topics may have been addressed by other international groups, include shared approaches to transdisciplinary scientific excellence and independence from vested political and commercial interests, enabling objective assessment of policy solutions.

### Multiple pathways of risk exposure and impact

In the present paper, we have selected topics for mitigation and adaptation covered in the regional phase of work to exemplify how the evidence base is being used by the academies to formulate their regional reports and associated contributions to inform policy discussions both national and international. In presenting this analysis, we also invite feedback from the wider scientific and medical communities to help guide our final synthesis and prioritisation of global issues. The topics addressed in the following sections were selected partly because of their importance to the region and relevance to other regions (acknowledging the need for contextual solutions as well as generalisability) but also by their applicability for informing policy making now.

The frequency, magnitude, and distribution of health risks depend on the nature of the hazard, the level of exposure to that hazard, and on personal/community vulnerability. IAP project analysis of the implications of climate change for health in all 4 regions supports the detailed assessments made by others [10,11]. Broadly, pathways of risk exposure can be characterised as direct (for example, heat and flooding), indirect via ecosystem disruption (for example, food and nutrition security, infectious disease incidence, and distribution), or indirect via socioeconomic system disruption (for example, declining labour productivity and increasing migration). Priorities for mitigation and adaptation with regard to health effects are described in the following sections.

### Health co-benefits of mitigation

Policies proposed to mitigate climate change provide global health benefits through reduced impacts and could also lead to localised improvements in the health of those populations undertaking the mitigation [12]. Parties to the Paris Agreement are required to include a mitigation contribution in their Nationally Determined Contributions (NDCs), and, in this context, it is important to take account of the large opportunities for public health gains [13]. Modelling scenarios analysing the health co-benefits of NDCs for selected countries in Africa, Asia, the Americas, and Europe [14] project a significant reduction of premature deaths related to air pollution, diet, and physical inactivity. Identifying health protection and improvement as priority outcomes in the NDCs requires (i) continuing commitment to measuring and monitoring the health co-benefits; (ii) ensuring policy coherence between climate change and health policy processes; and (iii) that health actions in the NDCs are comprehensive enough to build climate-resilient health systems [13]. Some of these mitigation priorities are summarised in Table 1. Some general principles for selecting climate change mitigation actions can be outlined, including consideration of the potential for cost effective GHG reductions and the resulting health (co-)benefits. Those countries who are the greatest GHG emitters should lead mitigation efforts, but there may be major differences in the sectoral contributions to GHG emissions, and quantification of effects and selection of the location and scale of solutions can still be challenging.

**Table 1:** Examples of mitigation actions: linking scientific evidence and policy objectives.

Policy objective	Examples of issues covered in regional academy work
Reducing anthropogenic air pollution through use of clean renewable energy sources	Fossil fuel combustion in high- and middle-income countries and burning of biomass in low-income countries account for high proportion of GHGs and airborne particulate pollution. Reducing fossil fuel use will slow climate change and bring major health benefits worldwide [15] including through reduction in lung cancer, cardiovascular, cerebrovascular, chronic obstructive pulmonary, and other noncommunicable diseases, e.g., in Europe [16], India, and China [17].
Sustainable cities and the built environment	About 75% of energy-related GHG emissions arise from urban activities. Significant health benefits can be obtained by providing accessible public transport and encouraging physical activity, as seen in New Zealand [18]; providing safe access to green space, such as the creation of “Superblocks” in Barcelona [19]; and improving housing insulation and ventilation [12]. However, mapping of urban case studies finds that cities in regions with highest mitigation relevance are systematically underrepresented [20]; increasing pace and scale of urban transformation requires changes in political, social, and economic systems [21].
Sustainable food systems	Agriculture and associated land conversion accounts for about 30% of GHG emissions worldwide [7,22]. The policy objective is to reduce malnutrition in all its forms while also reducing the contribution that food systems make to climate change [23]. Required carefully designed, evidence-based actions that are adapted to circumstances [24] include improving agronomic practices (such as regenerative agriculture), reducing waste, and increasing consumption of predominantly plant-based diets [7,25]. Coordinated action to counter the climate change effects on food systems is necessary to avoid unintended negative effects.

GHG, greenhouse gas.

### Challenges for adapting to health effects of climate change

Some examples of assessment of the scientific evidence are provided in Table 2, drawn from specific regions to illustrate the evidence, but in many cases, the issues are relevant worldwide. Our regional cross-cutting assessments are summarised as follows.

**Table 2:** Examples of regional climate change impacts on health with implications for adaptation.

What needs to be tackled?	Examples of issues covered in regional academy work
Extreme heat exposure (Americas)	Increased heat-related mortality is projected, regardless of the emissions scenario [33] with effects including heat exhaustion, heatstroke, cardiovascular challenges, renal failure, respiratory distress, and mental health effects, including suicide. The effects are not equally distributed and are dependent on local climates as well as demographic and socioeconomic factors [34].
Flooding (Asia-Pacific)	Current increases in rodent-borne (leptospirosis), waterborne (diarrhea), and vector-borne diseases associated with rainfall and temperature increases (for example, dengue [35]) are projected to worsen according to computational simulations of extreme weather events and urbanisation.
Wildfires (Asia-Pacific)	The very large scale of recent fires suggests that the limit to adaptation is being reached. Climate change may exacerbate the consequences of deliberate burning of forests and peatlands [6]. A detailed assessment of the early consequences of the 2019–2020 Australian bush fires reported health effects from direct exposure to flames and extreme heat, prolonged smoke inhalation, contamination of food and waterways, and through trauma [36].
Food and nutrition security (Africa)	Previous IAP work in Africa and elsewhere [9] reviewed the broad priorities for agriculture under climate change and the strategic importance of integrating activities for mitigation and adaptation, with co-benefits for health and development. Adaptation and low-carbon development pathways are priorities for the continent since it is largely paying for the consequences of high emissions elsewhere [37]. Solutions must also decouple, as far as possible, increases in livestock and crop productivity from GHG emissions [38], together with reducing waste and promoting sustainable consumption patterns [39].
Infectious diseases (Europe and Arctic)	Europe is susceptible to increases of some vector-borne diseases in humans (e.g., West Nile virus, Lyme disease, dengue, and chikungunya) and in livestock (e.g., African swine fever) under various climate scenarios [7]. There are also threats of increased waterborne (e.g., *Vibrio* species) and food-borne (e.g., *Salmonella* species) diseases. A rapidly warming Arctic (European and other countries) will increase threats for tick-borne diseases, malaria, West Nile virus, and *Vibrio* species, and the thawing permafrost may release anthrax [40] and previously unknown bacteria and viruses.

The evidence presented is from the region identified, but the issues are relevant for all regions.

GHG, greenhouse gas; IAP, InterAcademy Partnership.

#### Heat

Under high-emissions scenarios, large increases in heat-related deaths (and in net deaths including the possible effects of reduced cold exposure) are projected by late this century in warmer regions, such as the central and southern parts of America or Europe, and, particularly, Southeast Asia. There is a lack of data on which to base projections for Africa [26]. However, a recent systematic review of the United Kingdom literature [27] found that extreme heat exposure is often an invisible risk, whose impacts on health are not always recognised, and that there is insufficient policy action to prepare for direct or indirect effects of heat on health. Local actions, such as reducing heat island effects by better urban planning, including increasing green spaces, can reduce heat-related deaths. High temperatures also have other deleterious consequences, for wildfires, food systems, labour productivity, and pathogen distribution and replication that are discussed later.

#### Flooding

Flood risk is increasing around the world because of climate change and the interaction with socioeconomic development, including increased urbanisation and inadequate planning [28]. The various climate-induced causes include sea level rise, extreme weather events, excess precipitation, thawing permafrost, and melting glaciers, all of which are exacerbated by land-use changes, particularly urbanisation. There are multiple short-term health consequences such as accidental injuries or death, as well as waterborne and vector-borne diseases in some regions, linked to impaired quality of water for drinking and sanitation. There are also longer-term consequences resulting from ecosystem degradation, including chemical contaminants, as well as increasing mental health challenges and cardiovascular disorders, although more evidence is needed.

#### Wildfires

While fires can play a natural role in different ecosystems, they can also have a devastating long-term effect on ecosystems. Increasing temperatures and decreasing precipitation should be significant factors in the origin or increased frequency of wildfires worldwide, and a recent review has summarised the growing evidence linking climate change with increasing wildfire risk [29]. The ability to quantify and attribute the health consequences of pollutants from fires is challenging, but exposure to wildfire smoke is associated with increased all-cause mortality, and the particulate matter from wildfires may be more lethal than that from urban settings [30].

#### Food and nutrition security

Climate change is already adversely affecting the yield and quality of crops in many regions, and these impacts are compounded by other environmental changes, for example, soil and water degradation in consequence of added chemicals (fertilisers, pesticides, and herbicides), largely the result of industrial agriculture. The consequences for undernutrition, especially for vulnerable groups, will be compounded increasingly as heat- and humidity-induced declines in labour productivity affect growing numbers of subsistence farmers [31]. Furthermore, climate-related disruptions to local food systems have important implications for cultural continuity and livelihoods that underpin health and well-being, especially for Indigenous Peoples, farmers, and fishers who have close relationships with the environment [32].

#### Infectious diseases

A link between climate change and diverse infectious disease threats has been observed worldwide [2], in association with other aspects of globalisation that drive changes in both ecosystems and human behaviour. As a consequence of more favourable environments for mosquitos, WHO estimates that there will be significant increases in malaria and in other vector-borne viruses such as dengue, chikungunya, and Zika in some regions. Shifting distribution patterns of infectious diseases as a result of climate change are a global concern, both from increasing levels in locations where the diseases already exist and from their expansion into new areas.

The examples presented in Table 2 do not themselves determine how action should be focused or integrated across sectors and levels of governance. These issues are addressed further subsequently, but we emphasise here that integrated action, whether horizontal (across sectors) or vertical (between levels of governance), must not lead to any deflection of responsibilities.

### Using scientific evidence to identify and inform policy options worldwide

Policies for adaptation and mitigation are both needed for a systems-based approach [6] and must be better integrated across sectors and better linked with impact assessment and health guidance [4].

#### Heat

Current heat-related morbidity and mortality are partly preventable, given access to adequate infrastructure and appropriate policies [41]. However, adaptations cannot indefinitely keep pace with future warming, and some parts of the world may reach the limits to survival later this century under high-emission scenarios [31]. Solutions have focused on both short-term and longer-term technological, societal, institutional, economic, and behavioural interventions. These include appropriate building design, green infrastructure, and heat warning systems that trigger responses and resources to reduce the amount of time that people are exposed to extreme heat. However, the availability of these interventions is inequitable as some adaptation success to date is attributed to increased air conditioning and fans [41]. This underscores the importance of addressing adaptation and mitigation together, working across disciplines and sectors, and considering equity and social justice when developing and implementing heat-related health interventions.

#### Flooding

Cross-sectoral action is required. For built environments, this includes addressing priorities for urban planning, coastal defences (including river barrages), and resiting health facilities away from locations at risk of flooding. Responses must include nature-based solutions, such as wetland and mangrove restoration, as well as physical engineering measures, while taking into account the possibility of inadvertent health consequences (e.g., action to increase wetlands may provide new sites for disease vectors [7]). Flood-related policy initiatives and guidelines (including building standards, location of utilities, and medical facilities) often lack adequate consideration of future risk. Climate change and disaster risk management should be increasingly integrated, for example, as part of the Sendai Framework for Disaster Risk Reduction [42].

#### Wildfires

Action is needed at national and regional levels for cross-border pollution threats, including the following: (i) better public information on issues such as correct use of face masks and early warning systems, especially for vulnerable groups; (ii) research on the health consequences of exposure to different pollutants, as well as long-term follow-up on the mental health consequences of displacement and loss; (iii) integrating public health and healthcare services into disaster planning, especially for remote areas, using digital methodologies for early warning, monitoring, and delivery of health services; and (iv) increased national commitment to the conservation of forests and peatlands as carbon sinks and eliminating the use of fire to remove crop residues. This requires incentives and subsidies to recognise the value of diverse ecosystem services. It is also essential to have concerted international policy action to reduce consumer demand in developed countries for the commodities based on land clearance.

#### Food and nutrition security

There are many challenges to achieve climate-resilient pathways for food and nutrition security worldwide [8,9]. One key emerging issue is that current food policies in many countries concentrate more on how to protect consumer health from contaminated food than the degree to which the state should use health and environmental considerations to regulate the marketing of food items [39]. Government policies, for example, can support rebalancing consumption by introducing incentives/disincentives (dietary guidelines, food labelling, pricing, and taxation) for healthy, sustainable dietary choices, while protecting vulnerable groups.

#### Infectious diseases

Early warning systems and other interventions have high intersectoral relevance because they both improve public health and help sustain economic output that is otherwise reduced by the consequences of infectious disease outbreaks disrupting travel and work. Opportunities and challenges for worldwide policy integration have been accentuated by the Coronavirus Disease 2019 (COVID-19) pandemic, which has exerted very large pressures on the health sector and revealed a lack of preparedness at many levels in most countries. The impacts of climate change will increasingly add pressures on health systems. In addition, both climate change and COVID-19 have very high public health and economic impacts, exerting disproportionate effects on vulnerable groups. The effects of climate change and COVID-19 may interact in various ways, for example, climate change–induced flooding has undermined public health responses to COVID-19, and the impact of COVID-19 on food systems may compound vulnerabilities in low-income groups [43]. In policy terms, these and other mutually reinforcing adverse consequences of climate and COVID-19 crises underpin the importance of progressing coordinated policy for sustainable recovery after COVID-19 [44].

## Conclusions

Many policy solutions are advanced at a national level, including adaptation in health and social systems: Now, it is increasingly important to take account of the health implications for climate change policy integration in all sectors and to bring the interventions to scale [1]. We emphasise that there must be more commitment from policy makers to integrate health into National Adaptation Plans and NDCs of GHG emission reductions under the Paris Agreement. In addition to required national actions, health policy objectives must have regional considerations when there are cross-border threats emerging such as air pollution or infectious diseases. Pollution from industrial, urban, or agricultural origin near to national borders can have major consequences for neighbouring countries (for example, in the Indian subcontinent [45]). Interregional implications (spillover effects) may also be relevant, if national or regional policy action in one area, inadvertently or not, leads to adverse consequences elsewhere. For example, many nations are currently exporting their lack of environmental sustainability by importing food/biomass generated unsustainably elsewhere. Competition between food, feed, and fuel for the effective use of natural resources requires consideration of multiple factors in understanding trade-offs and setting priorities [46]. Protection of natural resources is also highly relevant in the search for natural climate solutions. Specific examples (in flood protection and for forest conservation as a carbon sink) have already been mentioned, and there is now a significant evidence base [47] from solution-oriented research on natural options in both mitigation and adaptation with benefits for health. In considering coordinated responses to environmental risks, it is essential to tackle the challenges for climate change and biodiversity together [48].

In addition to national and regional policy action, there is considerable scope for integration at the global level. Some of the current policy opportunities for embedding adaption and mitigation solutions worldwide, to underpin the objectives for health, social justice, sustainability, and survivability, include collective action on the Sustainable Development Goals (SDGs), the current discussions of the UN Framework Convention on Climate Change (UNFCC) and UN Convention on Biological Diversity (CBD), and regional initiatives by the offices of other UN bodies, in particular WHO, Food and Agriculture Organization (FAO), and UNEC (United Nations Economic Commission). Action is also possible as part of G7 and G20 Presidency’s initiatives and when linked to other strategic initiatives in pursuit of the circular economy and bioeconomy. In all cases, there are crucial roles for the scientific community in advising on the opportunities for science and technology, connecting to innovation, policy, and practice. Coherent policy development requires both vertical integration (local–regional–global) and horizontal integration between sectors, and both can be consolidated through the SDG framework. However, this requires careful planning. For example, although decarbonisation measures can benefit multiple SDGs, poorly designed ones may incur harm if generating resource conflicts or excluding communities [49]. There is concern that, as decarbonisation goals are increased to meet the Paris Agreement targets, so does the potential for adverse social outcomes, particularly increased inequity. To avoid these negative consequences, it is vital to clarify and agree objectives for triple wins on environment, health, and equity.

In the past decade, there has been a surge of international agendas to address global challenges: A focus on human health helps to catalyse the strengthening and linkage of these agendas [42] in driving national and regional policy decisions. The global context is important to strengthen and safeguard those goods and services that are provided on a scale beyond countries and can only be achieved collectively, for example, GHG emissions reductions, rule-based trade, food and nutrition security, pandemic prevention and management, and shared science. Mitigation measures may be initiated at local or regional level but require globally shared commitment to decarbonisation, with the imperative for the high emitters to act decisively. Shared objectives require collective action to tackle environmental and institutional risks and their transmission in an uncertain and rapidly connected world. Hitherto, policy making has often focused on reactive strategies but must now be more anticipatory, based on robust evidence, and with commitment to monitoring and auditing impacts to document the accumulating experience to improve future policy. While there are evidence gaps to be filled by new research, these should not be used as an excuse to delay action in developing policy on the basis of the best available science.

There is great heterogeneity within and between regions, but the findings of IAP’s regional-to-global projects confirm the potential value in sharing knowledge that will inform policy and practice worldwide. Utilising diverse evidence streams from the regional assessments necessitates embracing and supporting both more quantitative and qualitative research, the latter to understand the lived experiences of climate change impacts on health outcomes, as well as the contexts within which adaptation and mitigations efforts unfold. For example, other recent work on climate change and health issues for Arctic populations and their neighbours by IAP together with EASAC and the US National Academies of Science Engineering and Medicine [50] included discussion of impacts on Indigenous People, their contribution to transdisciplinary research efforts, and the value of traditional knowledge.

One research priority is to better understand the barriers limiting the implementation of action on climate change. Dismantling these barriers partly depends on effective public engagement by the scientific and health communities to articulate both the health threats associated with climate change and the solutions to the threats. Improving awareness about the health implications and countering misinformation can be a potent way to encourage public willingness to mitigate climate change through changing behaviour [7]. Another barrier to implementation rests on assumptions about the high economic costs of change. To what extent does perception of immediate economic costs inhibit the influence of health co-benefits on the development of mitigation policies [5]? There is evidence to suggest that including health in the cost-benefit analysis of mitigation measures strengthens the case for setting and meeting ambitious policy targets [7]. Further studies on cost-effectiveness of health effects are warranted to challenge the perception that change necessarily entails high costs and to contribute to the broader, transdisciplinary, multi-sectoral evidence base on the costs of action and inaction [51,52].

The political momentum for tackling climate change must accelerate, and there are significant upcoming opportunities to inform and influence public policy. For example, health issues are highly relevant to the 2021 UK Presidency themes for the Conference of the Parties (COP26) of the UNFCCC (adaptation and resilience; energy transitions and natural habitats; clean transport; cities and the built environment; and financial systems; see https://ukcop26.org). As noted in the Introduction, the scientific and health communities have important roles to play in these discussions, leading by example with actions in the health sector itself [53], and, more broadly, to share lessons of good practice, build capacity for action worldwide, and ensure that robust evidence is generated. But this is not enough, there is also a collective responsibility to use the principles of co-design with stakeholders to help build user receptivity at the science–policy interface as part of integrated goals to inform policy development, public engagement, and transformation of systems.

## Supporting information

S1 Text Regional working groups’ composition and procedures. (DOCX)

## Abbreviations

AASSA: Association of Academies and Societies of Sciences in Asia
CBD: Convention on Biological Diversity
COP15: Conference of the Parties 15
COP26: Conference of the Parties 26
COVID-19: Coronavirus Disease 2019
EASAC: European Academies’ Science Advisory Council
FAO: Food and Agriculture Organization
GHG: greenhouse gas
IANAS: InterAmerican Network of Academies of Sciences
IAP: InterAcademy Partnership
LMIC: low- and middle-income country
NASAC: Network of African Science Academies
NDC: Nationally Determined Contribution
SDG: Sustainable Development Goal
UNFCCC: United Nations Framework Convention on Climate Change
