# Supplementary material for: Evidence-informed policy for tackling adverse climate change effects on health: Linking regional and global assessments of science to catalyse action
Source: PLoS Med. 2021 Jul 20;18(7):e1003719. doi: 10.1371/journal.pmed.1003719 (PMC8330928; doi:10.1371/journal.pmed.1003719)
Supplement: S1 Text — (DOCX) [file pmed.1003719.s001.docx]

S1 Text for PLoS Medicine

Regional working groups’ composition and procedures

Country involvement in regional academy network working groups

Africa: Benin, Botswana, Burkina Faso, Burundi, Cameroon, Cote D’Ivoire, Ethiopia, The Gambia, Kenya, Mauritius, Nigeria, Senegal, South Africa

Asia-Pacific: Armenia, Australia, Azerbaijan, Bangladesh, China, India, Indonesia, Japan, Malaysia, Nepal, New Zealand, Pacific Islands, Pakistan, Republic of Korea, Russian Federation (Far East), Turkey

Americas: Argentina, Brazil, Canada, Costa Rica, Guatemala, Mexico, Peru, United States

Europe: Cyprus, Czech Republic, Finland, Germany, Greece, Ireland, The Netherlands, Poland, Portugal, Sweden, Switzerland, UK

Scientists from additional countries are involved in peer review.

Each expert group used a common template of questions to scope observed and projected effects of climate change on health, as well as potential solutions for both adaptation and mitigation. They are examining priorities for the region, taking account of diversity in climate effects, research and health system capacities, and development status.

The IAP project, initiated in November 2019, used a prior assessment of the situation in Europe to provide the common starting points for the other regions. The peer-reviewed, consensus regional reports for Africa, Asia and the Americas will be published during 2021. Regional outputs are being brought to the attention of national and regional policy makers, for example as part of activities in preparation for COP26, as well as other stakeholders through dissemination and engagement activities. In addition, they are being used as a resource for a global report, to be published in 2022. This report will examine similarities and differences between regions to inform the science-based policy focus on inter-regional and global priorities.
